# Editorial: Plant nutrient use efficiency in the era of climate change

**DOI:** 10.3389/fpls.2024.1402868

**Published:** 2024-04-10

**Authors:** Arun K. Shanker, Lekshmy Sathee, Vanita Jain, Nandula Raghuram

**Affiliations:** ^1^ Division of Crop Sciences, Indian Council of Agricultural Research (ICAR)-Central Research Institute for Dryland Agriculture, Hyderabad, India; ^2^ Division of Plant Physiology, Indian Council of Agricultural Research-Indian Agricultural Research Institute (ICAR-IARI), New Delhi, India; ^3^ Agricultural Education Division, Indian Council of Agricultural Research (ICAR)-KABII, New Delhi, India; ^4^ Centre for Sustainable Nitrogen and Nutrient Management, USBT, GGSIPU, New Delhi, India

**Keywords:** climate change, plant nutrition, elevated (CO_2_), nitrogen nutrition, phosphate-based fertilizers

The critical need for climate resilience is more evident now than ever, with the shadow of climate change casting a vast uncertainty over our future. This urgency intersects notably within agriculture, where the dual goals of achieving food security for an expanding global populace and adopting sustainable production practices are paramount. Central to sustainable farming is the efficient utilization of nutrients, especially nitrogen, given its profound influence on both crop productivity and environmental well-being. The complexities of nutrient management are intensified by climate change, with unpredictable weather, rising temperatures, and shifting precipitation patterns impacting nutrient absorption by crops and the effectiveness of fertilizers. Thus, optimizing nutrient management transcends improving yields; it’s about fortifying agriculture against climate-induced adversities.

Recent technological strides in agriculture have zeroed in on improving nutrient efficiency, marking a pivotal moment in research amidst escalating climate and environmental challenges. Research must now focus on fine-tuning nutrient delivery, considering the precise needs of different crops under evolving weather conditions, while prioritizing soil and water conservation, and lowering greenhouse gas emissions. Economically, making these innovations affordable and scalable for farmers is essential. However, the scalability, cost, and farmer accessibility of such innovations, particularly in less developed regions, require careful consideration. Adapting these technologies to varied crops and climates poses additional challenges. This editorial encapsulates the essence and implications of recent findings on nutrient efficiency and climate resilience, advocating for a future where advanced tech meets sustainable agriculture to secure food in an eco-conscious manner.

The article by Bhavya et al. presents a nuanced understanding of how elevated CO_2_ levels impact rice cultivation, with a particular focus on yield, quality, and nutrient content. Under increased CO_2_ conditions, there was a noted increase in the number of tillers, yet this was accompanied by a decrease in both the number of grains per panicle and overall grain yield, attributed primarily to the effects of higher temperatures. Additionally, elevated CO_2_ led to an increase in dry matter production and straw yield. In terms of grain quality, the study found that while carbohydrate content in grains increased under elevated CO_2_, there was a concurrent decrease in both protein and amylose content. This alteration in nutritional composition underlines the complexity of CO_2_’s impact on crop quality.

A critical aspect of the study was its focus on the iron content in rice. Elevated CO_2_ levels were observed to reduce iron content, uptake, and accumulation in rice plants at both pre-anthesis and post-anthesis stages, culminating in lower iron levels in the rice grains and bran. The study contributes valuable knowledge to the ongoing discourse on agricultural practices in the face of changing climatic conditions, emphasizing the need for innovative strategies to sustain crop quality and nutritional value.

Wang et al. have conducted a comprehensive investigation into how elevated carbon dioxide (CO_2_) levels and canopy warming affect nutrient status and dynamics in wheat cultivation. The findings of the study are particularly enlightening with respect to the impact of canopy warming, which was found to have a more pronounced effect on nutrient concentrations and translocation than elevated CO_2_ alone. Canopy warming notably increased nitrogen, phosphorus, and potassium concentrations in wheat shoots, while simultaneously reducing these concentrations in the roots. This phenomenon was further characterized by a significant increase in the transfer of nutrients from roots to shoots, as reflected in higher nutrient transfer coefficients. In contrast, elevated CO_2_ in isolation had a more subdued effect. However, under ambient CO_2_ conditions, canopy warming was observed to increase the availability of soil nitrate and phosphorus. In summary, this study illuminates the dominant influence of canopy warming on nutrient dynamics and uptake in wheat, suggesting that wheat plants might demand more nutrients under future warming scenarios.

The work of Hibbert et al. delves into the cultivation of watercress, a nutritious leafy green crop ideally grown in aquatic environments, and examines the environmental implications of using phosphate-based fertilizers. The excessive use of these fertilizers in watercress farming can lead to pollution and the eutrophication of aquatic habitats, especially in chalk streams. The research focused on understanding the effects of reducing phosphate fertilizer application on the morphology, biochemistry, and gene expression of watercress. Findings from the study revealed that watercress plants could sustain their shoot yield even without additional fertilizer. RNA sequencing provided insightful data, indicating that genes related to cell membrane remodeling, root development, and phosphate transport were differentially expressed under low fertilizer conditions. Interestingly, two specific watercress lines, numbered 60 and 102, demonstrated distinct strategies for tolerating low phosphate levels, positioning them as promising candidates for future breeding efforts. Notably, the reduction in phosphate fertilizer application did not adversely affect the glucosinolate concentrations in watercress, which are vital for its nutritional value.

Bana et al. in their work present an in-depth analysis of the impact of various groundnut-based cropping systems and nutrient management practices on crop productivity, soil health, and economic returns in a semi-arid region of South Asia. One of the study’s notable findings was the superior performance of the Groundnut-marigold cropping system in terms of both system productivity and net returns. This success was attributed to the higher market value of marigold compared to wheat and fenugreek. The most successful strategy identified in the study was the combination of the Groundnut-marigold cropping system with the application of leaf compost and 50% of the recommended chemical fertilizers during the wet season, followed by the full recommended dose in the dry season. Such strategies can substantially improve crop yields, enhance farm incomes, and promote soil health in a sustainable manner.

Lv et al. investigated the effects of various forms of foliar-applied nitrogen (NH_^4+^-_N, [NO_3^-^_, NH_4^+^_, and CO(NH_2_)_2_] on wheat grain filling and starch accumulation under drought stress. They found that different nitrogen sources had distinct impacts on grain filling dynamics, with NH_4_+-N prolonging the grain filling period, whereas NO_3_−-N and CO(NH_2_)_2_ accelerated it and improved starch synthesis by influencing gene expression related to the sucrose-starch conversion pathway. The study suggests that foliar nitrogen application, especially NH_4_^+^-N, enhances the antioxidant enzyme system and modulates phytohormone levels, thereby mitigating drought stress effects and potentially improving wheat productivity under water-limited conditions.

Overall, the significance of the articles in the Research Topic emphasizes the uncertainties of climate change, the fusion of technology, agriculture, and environmental stewardship as paramount for the future. The commendable efforts in enhancing plant nutrient use efficiency must evolve into a comprehensive strategy that embraces economic, environmental, and social facets to foster sustainable agricultural practices. Future research directions include developing climate-resilient crops through genetic and biotechnological innovations, integrating smart farming technologies for precision agriculture. This will deepen our understanding of soil-microbe-plant dynamics, alongside fostering interdisciplinary collaborations to ensure the practical application of these advancements aligns with the multifaceted challenges of climate change.

## Author contributions

AS: Writing – original draft, Writing – review & editing. LS: Conceptualization, Project administration, Writing – review & editing. VJ: Conceptualization, Supervision, Writing – review & editing. NR: Conceptualization, Writing – review & editing.

